# Impact of a single, short morning bright light exposure on tryptophan pathways and visuo- and sensorimotor performance: a crossover study

**DOI:** 10.1186/s40101-018-0173-y

**Published:** 2018-04-23

**Authors:** Wolfgang Schobersberger, Cornelia Blank, Friedrich Hanser, Andrea Griesmacher, Markus Canazei, Veronika Leichtfried

**Affiliations:** 1 Institute for Sports Medicine, Alpine Medicine and Health Tourism, UMIT - University for Health Sciences, Medical Informatics and Technology, Eduard Wallnöfer Zentrum 1, 6060 Hall in Tyrol, Austria; 2 Tirol Kliniken GmbH, Anichstraße 35, 6020 Innsbruck, Austria; 3 Department of Biomedical Computer Science and Mechatronics, Institute of Electrical and Biomedical Engineering, UMIT - University for Health Sciences, Medical Informatics and Technology, Eduard Wallnöfer Zentrum 1, 6060 Hall in Tyrol, Austria; 4 Central Institute of Medical and Chemical Diagnostics, LKH – University Hospital of Innsbruck, Anichstraße 35, 6020 Innsbruck, Tyrol Austria; 5 Department of Visual Perception, Bartenbach GmbH, Rinner Strasse 14, 6071 Aldrans, Tyrol Austria

**Keywords:** Bright light, Sensorimotor performance, Visuomotor performance, Tryptophan, Melatonin, Kynurenine

## Abstract

**Background:**

Bright light (BL) has been shown to be effective in enhancing both cognitive and physical performances. Alterations in nighttime melatonin levels have also been observed. However, evaluations of light-induced changes in the preceding biochemical processes are absent. Therefore, the impact of a single morning BL exposure on sensorimotor and visuomotor performance, as well as tryptophan (trp) and trp metabolites, was evaluated in this study.

**Methods:**

In a crossover design, 33 healthy volunteers were randomly exposed to 30 min of < 150 lx at eye level (office light, OL) and 5000 lx at eye level (bright light, BL) of 6500 K in the morning hours. Trp, sulfatoxymelatonin (aMT6s), and kynurenine (kyn) courses over the morning hours were analyzed, and changes in sensori- and visuomotor measures were examined.

**Results:**

Motoric performance increased in both setups, independent of light intensity. aMT6s and kyn decreased equally under both lighting conditions. Trp levels decreased from a mean (95% confidence interval) of 82.0 (77.2–86.9) to 66.5 (62.5–70.1) in the OL setup only.

**Conclusion:**

These data suggest that BL in the morning hours has a limited effect on visuo- and sensorimotor performance. Nevertheless, trp degradation pathways in the morning show diverse courses after OL and BL exposure. This suggests that trp courses can potentially be altered by BL exposure.

## Background

Even without environmental stimuli, both cognitive and physical performance vary in an iterative rhythm lasting approximately 24 h. Especially in the morning hours, performance is reduced [1, 2]. However, optimized performance and productivity are often desired regardless of the time of the day (i.e., for sport competitions and in the work environment). Therefore, a demand for performance-enhancing strategies exists.

The suprachiasmatic nuclei (SCN) play an important role in diurnal variations of mental and physical performance and strongly determine productivity and error-susceptibility [3, 4]. SCN activity can be modulated by exogenous parameters, e.g., sleep-wake patterns, meals, activity, or light exposure.

Light plays an essential role in modulating the circadian system either by direct effects or phase-shifting effects on the circadian clock. Optimized indoor illumination and bright light (more than 1000 lx at eye level) were shown to be of value for health and work efficiency strategies, inducing positive effects on mood and increasing activation [5–8], as well as cognitive and exercise performance [9–12].

One underlying mechanism might be light’s ability to influence levels of hormones that vary approximately with a period length of 24 h, such as melatonin and its precursors [13–15]. Tryptophan (trp) is the basic molecule that can be converted to serotonin and further degraded to melatonin [16]. Advancement of the circadian phase via morning bright light (BL), in combination with afternoon melatonin uptake, recently proved successful [17]. Numerous studies have reported the impact of light exposure on melatonin production and excretion, with an emphasis on its relevance for cognitive and physical performance [18, 19]. Beyond acute nighttime suppression of melatonin, there are several studies regarding the impact of BL on alertness [1, 20], as well as cognitive [18, 21, 22] and physical performance [22–27]. However, research specifically focusing on the potential consequences of acute BL application on sensorimotor (coordination) and visuomotor (reaction) performance is lacking. Moreover, research on potential underlying biochemical mechanisms of BL, such as melatonin precursors and trp metabolites, known collectively as kynurenines (kyns), which are involved in a variety of biological activities (e.g., inflammation, immune response, exercise, mental health) [28], is scarce [29, 30]; addressing this gap could augment our understanding of the impact of BL on cognitive and physical performance and its biochemical pathways.

The aim of this study was twofold. First, we evaluated the impact of a single 30-min BL exposure in the early morning hours on visuomotor and coordinative performance in healthy adults. Second, we collected data on trp and trp metabolites (kyn) to assess the potential association between BL-induced changes in visuomotor and coordinative performance and changes in trp and trp metabolites. It was hypothesized (H1) that visuomotor and coordinative performance would improve after BL exposure. Furthermore, it was hypothesized (H2) that urine melatonin levels would be suppressed because of the morning BL exposure. Finally, we also hypothesized (H3) a significant negative association between changes in visuo- and sensorimotor performance and trp, as well as kyn.

## Materials and methods

### Participants

Via the Internet, 35 study participants between 18 and 45 years of age, from the Tirol Kliniken/University Hospital Innsbruck (Austria), the University for Health Sciences, Medical Informatics and Technology (UMIT, Hall, Austria), and the Tyrolean Confederation of Sports Shooters (Austria), were recruited. None of the participants were on medications, had ever worked in shifts, had taken a transmeridian flight in the previous 2 months, or suffered from chronic diseases. Moreover, all participants maintained their daily lifestyles including regular sleeping times at least 2 weeks before the study begins. For details, see Leichtfried et al. [20].

Additionally, respondents with depressive and/or sleeping disorders were excluded from the study. To evaluate depression and depressive symptoms, the Hospital Anxiety and Depression Scale (German version; HADS) was used by applying a cut-off value > 8 as the exclusion criterion [31]. Sleep quality was determined via the Pittsburgh Sleep Quality Index (PSQI) by applying a cut-off value > 5 as the exclusion criterion [32]. All participants gave written informed consent prior to participation in the study.

### Experimental trials

A prospective, explorative crossover design with a washout phase of 7 days was implemented. In order to control for natural daylight, all investigations were performed in the winter months (15 November–25 February). On both test days, the participants slept at their homes, woke at 6:00 a.m., collected their first morning urine samples (all participants were instructed how to provide urine samples), and had their habitual breakfast. There was no instruction regarding a standardized breakfast, but all participants were told to follow their usual morning routine. Participants arrived at the study location at 7:00 a.m. and brought their first morning urine samples along with them. During the study period, the sun had not risen at the start of the indoor protocol at 07:05 a.m. (November 15: 07:18 a.m.; December 1: 07:40 a.m.; January 1: 08:01 a.m.; February 1: 07:41 a.m.; February 25: 07:07 a.m.), thus guaranteeing that bright light exposure by sun light had not affected the volunteers at the start of the indoor protocol at 07:05 a.m. On two occasions, according to an interval of 7 days, each study participant was exposed to light for 30 min starting at 07:40 a.m. One of the exposures was to BL and the other to office light (OL). Before and after the light exposures, participants stayed in the same room, illuminated by normal workplace light (< 100 lx at eye level, 4000 Kelvin). In total, 18 of the participants were randomly assigned to receive the BL exposure first, followed by the OL exposure 7 days later, and the other 17 participants were assigned to receive the OL exposure first, followed by the BL exposure 7 days later. Identical experimental procedures on both days of exposure were applied (Table 1).

**Table 1:** Experimental procedure on both study days

Time of day (a.m.)	Activity	Light exposure
06:00	Wake up (morning urine collection)	Residential light
07:00	Arrival at the study location	Workplace
07:05	Urine sampling I, blood sampling I	Workplace
07:15	VAS pre (data not shown, [26])	Workplace
07:20	Visuo- and sensorimotor performance tests I	Workplace
07:40–08:10	Light exposure	BL or OL
08:12	VAS post (data not shown, [26])	Workplace
08:15–09:00	Sustained attention test (data not shown, [26])	Workplace
09:05	Urine sampling II, blood sampling II	Workplace
09:15	Visuo- and sensorimotor performance tests II	Workplace
11:05	Urine sampling III, blood sampling III	Workplace

*VAS* visual analogue scale, *pre* before light exposure, *post* after exposure to either bright light (BL, 5000 lux, 6500 K) or office light (OL, < 150 lux, 6500 K); workplace light exposure < 100 lux, 4000 K

### Light exposures

Exposure to BL was performed in a light cabin [33], which was equipped with fluorescent lamps (OSRAM 58W/965 Biolux, 3700 lm) with a light intensity (illuminance) of 5000 lux at eye level, a color temperature of 6500 K, and a mean field of view luminance of 1500 cd/m^2^. Without fixing the head and gaze of the subjects, corneal light levels could be kept constant in this cabin.

The photometrical specification of the OL setup, again performed in the light cabin, was chosen to be similar to conventional office illumination, with a light intensity of < 150 lux at eye level, and a mean field of view luminance of 85 cd/m^2^.

Table 2 summarizes relevant photometrical measures of both lighting scenarios according to the recommendations from Lucas et al. [34]. In terms of melanopic illuminance levels, BL was more than 33 times brighter than OL. For further specifications of the light cabin, see Leichtfried et al. [33].

**Table 2:** Photometrical measures of the light exposures

Photometrical measures [unit]	Office light (OL)	Bright light (BL)
Corneal intensity-related measures		
Photopic illuminance [lux]	150 ± 32	5000 ± 455
Irradiance [μW/cm^2^]	59.18	1972.53
Photon flux [1/cm^2^/s]	1.61 × 10^14^	5.37 × 10^15^
Photopigment-related measures (α-opic lux) [34] at mean illuminance levels		
Cyanopic illuminance [lux]	160.73	5357.65
Melanopic illuminance [lux]	152.88	5096.14
Rhodopic illuminance [lux]	154.17	5138.83
Chloropic illuminance [lux]	153.19	5106.38
Erythropic illuminance [lux]	147.36	4912.04

Illuminance values: mean ± standard deviation

Participants were instructed to keep their eyes open during OL and BL exposure. They were allowed to read newspapers, which were supplied by the authors.

Motor performance measures and blood parameters were recorded under standard workplace lighting from ceiling luminaires (equipped with fluorescent lamps; OSRAM 36W 840 Lumilux) with a light intensity of < 100 lux and a correlated color temperature of 4000 K at eye level of the study participants.

### Evaluation of chronotypes

Since the individual chronotype might influence the study outcomes, chronotypes of the participants were evaluated at the beginning of the study using the Morningness-Eveningness Questionnaire (MEQ) [35]. Analyses of biochemical parameters were controlled for chronotype.

### Physiological measures

Urinary 6-sulfatoxymelatonin (aMT6s) was assayed using a commercially available competitive immunoassay kit (Buehlmann 6-SMT ELISA, Buehlmann Laboratories AG, Schoenenbuch, Germany) with a lower detection limit of approximately 0.5 pg/mL (for details, see [5]). All aMT6s analyses were done at the Central Institute for Medical and Chemical Laboratory Diagnostics, Innsbruck, Austria, and creatinine (crea)-standardized to account for differences arising from variations in urine concentrations.

Serum trp and kyn concentrations were measured by high-pressure liquid chromatography (HPLC) using 3-nitro-L-tyrosine as an internal standard [36]. Reference values for healthy subjects are 67.4 ± 10.2 μmol/L for trp and 1.78 ± 0.42 μmol/L for kyn [37].

The following three physiological parameters were subjected to statistical analyses in the present study: urinary 6-sulfatoxymelatonin normalized for crea (aMT6s/crea), trp, and kyn.

### Performance tests

#### Visuomotor reaction time analyses

Reaction tests were performed using specifically designed equipment (Dr. Wieser GmbH, Salzburg, Austria) with four sensing devices, one for each hand and foot. Sixty random signals were transmitted to a rectangular black board (positioned in front of the participant) equipped with four red light-emitting diodes (LEDs) positioned on each corner of the black board. The participants were allowed to individually position the sensing devices (foot and hand switches) and were instructed to react as quickly as possible by touching the appropriate sensing device (top right—right hand, top left—left hand, bottom right—right foot, bottom left—left foot), depending on which LED flashed red. In addition to the total reaction time (TRT), the following four performance measures were recorded: number of correct reactions (CR), number of false reactions (FR), reaction time of correct reactions (RTCR), and reaction time for false reactions (RTFR). A test phase lasting 1 min was performed prior to the initial test.

#### Sensorimotor (balance) test

Balance tests were performed on an unstable surface (MFT Challenge Disk, MFT–Multifunktionale Trainingsgeräte GmbH, Guntramsdorf, Austria). Three-dimensional deviations from the horizontal zero position were evaluated using acceleration/inertial sensors. Sensors measure deviations from the horizontal plane, expressed as *A*_*z*_, reflecting the extent to which the unstable surface is tilted. Values increase with increasing deviation from the horizontal plane, and low *A*_*z*_ values reflect a “stable” balance.

A test phase of 1 min preceded every trial. After the test phase, the participant stepped off the unstable disc for 2 min and stepped on again for the actual recording phase, which lasted 2 min.

Balance tests were performed before and after light exposures, directly after the visuomotor reaction time analyses.

### Statistical analyses

The distribution of the data was evaluated using a graphical approach (boxplots). Parametric tests were used for normally distributed data and non-parametric tests for skewed data (aMT6s/crea).

Differences between the three chronotypes, for changes over time (∆pre-post), were evaluated via the Kruskal-Wallis test for unmatched samples.

The statistical approach was chosen according to recommendations for cross-over designs [38]: to eliminate possible carry-over effects, an initial test was done. The results for both study periods were calculated and added up, followed by an unpaired *t* test for the different sequence groups. A non-significant result indicates no carry-over effects. Treatment effects were calculated by comparing the respective differences between the measurement time points by performing an unpaired *t* test for the two sequence groups. For aMT6s/crea data, a Mann-Whitney *U* test was applied for both tests.

Regarding the biochemical parameters, changes over time in the two setups were calculated via linear models for repeated measurements and Friedman ANOVA (aMT6s/crea), including an interaction term (time × light). Dependent on the data distribution, the locations of possible changes were evaluated via *t* test and Wilcoxon’s test for paired samples (aMT6s/crea), with the Bonferroni correction applied.

To test for associations between changes in performance and biochemical parameters, Pearson (non-skewed data) or Spearman (skewed data) correlation analysis was performed as applicable. Even though data from four time points were recorded, deltas of change resemble the difference between biochemical parameters in the first and final urine sample taken. Effect sizes were presented as correlation coefficients, r, which followed Cohen’s convention for effect size classification.

Results from categorical variables were reported as proportions, and continuous variables were reported as means ± standard deviation (SD) or means and 95% confidence intervals (CI). All statistical analyses were performed using SPSS (Statistical Package for Social Sciences, ver. 20.0; SPSS Inc., Chicago, IL, USA). All statistical tests were performed at a 0.05 level of significance.

## Results

### Participants

Thirty-three participants (16 males, 17 females) with an average age of 33.0 ± 7.2 years were included in the analyses. Two volunteers were excluded during the study because of acute infections. The participants showed a mean PSQI score of 3.8 ± 1.8 and a sleep efficiency of 85%; no participant reported taking soporifics in the 4 weeks prior to the first light exposure. With respect to chronotype, 15.2% (5) of the participants were morning type, 12.1% (4) were evening type, and 72.7% (24) were moderate chronotype subjects. The results of the HADS-D indicated that 31 (93.9%) of the participants were not depressed (score < 7) and 2 (6.1%) of the participants showed signs of slight depression (score 8–10). Chronotype-dependent differences in change scores (∆pre-post) were detected for aMT6s values only (*p* = .009); thus, such differences were further considered only for aMT6s values.

### Performance data

#### Visuomotor performance

Carry-over effects for all parameters could be ruled out (CR, *p* = .862; TRT, *p* = .193; RTCR, *p* = .183; RTFR, *p* = .795; FR, *p* = .862). TRT ranged from 0.46 to 0.88 s. No changes in RTCR were detected at either light intensity. After both light exposures, CR was significantly higher and FR significantly lower as compared to before light exposure. No effects of light intensity were detected for any of the reaction parameters. Details are outlined in Table 3.

**Table 3:** Results of performance tests before and after office light (OL) and bright light (BL) exposure

	OL exposure			BL exposure			*p* value (OL vs. BL)
	Before	After	*p* value	Before	After	*p* value	
Visuomotor performance							
CR [*n*]	55.1 ± 4.3	56.6 ± 2.6*	0.021	55.6 ± 3.0	56.7 ± 2.0*	0.033	0.739
TRT [s]	0.63 ± 0.09	0.64 ± 0.08	0.803	0.63 ± 0.08	0.63 ± 0.08	0.975	0.959
RTCR [s]	0.64 ± 0.09	0.64 ± 0.08	0.843	0.64 ± 0.08	0.64 ± 0.08	0.706	0.813
RTFR [s]	2.45 ± 1.62	1.96 ± 0.37	0.223	2.16 ± 0.64	2.32 ± 1.54	0.626	0.128
FR [*n*]	4.9 ± 4.3	3.4 ± 2.6*	0.021	4.4 ± 3.0	3.3 ± 2.0*	0.033	0.739
Sensorimotor performance							
Az [m/s^2^]	0.60 ± 0.27	0.47 ± 0.17*	0.028	0.53 ± 0.17	0.47 ± 0.18*	0.005	0.275

Values represent mean values ± standard deviations

*OL* office light, *BL* bright light, *A*_*z*_ deviation from horizontal plane, *CR* correct reactions, *TRT* total reaction time, *RTCR* reaction time correct reactions, *RTFR* reaction time false reactions, *FR* false reactions

*p < 0.05 compared to before light treatment

#### Sensorimotor performance

No carry-over effects were detected (*p* = .884). Deviations from the horizontal plane (*A*_*z*_) ranged from 0.21 to 1.58 m/s^2^, with mean values of 0.52 ± 0.21 m/s^2^. Under DOL, *A*_*z*_ decreased from 0.60 ± 0.27 to 0.47 ± 0.18 m/s^2^ (*p* = .028). *A*_*z*_ also decreased from 0.53 ± 0.17 to 0.47 ± 0.17 m/s^2^ after BL exposure (*p* = .005). No effects of light intensity on balance performance could be found (*p* = .275). Details are outlined in Table 3.

### Biochemical data

All biochemical data are shown in Table 4. No carry-over effects were detected for any biochemical parameter.

**Table 4:** Sulfatoxymelatonin (aMT6s/crea), tryptophan (trp), and kynurenine (kyn) concentrations under office light (OL) and bright light (BL) exposure

Time	aMT6s/crea [ng/mg]		trp [μmol/L]		kyn [μmol/L]	
	OL exposure	BL exposure	OL exposure	BL exposure	OL exposure	BL exposure
Morning urine	20.8 (12.2–29.4)	14.0 (8.6–19.4)	n.a.		n.a.	
7:15 a.m.	17.8 (10.9–24.7)	14.6 (8.7–20.5)	82.0 (77.2–86.9)	75.6 (70.3–81.0)	3.2 (2.8–3.7)	2.8 (2.5–3.0)
9:15 a.m.	12.1 (8.0–16.1)^#^	12.2 (7.3–17.1)^#,^*	69.5 (65.0–74.1)*	69.3 (64.9–73.7)	2.8 (2.6–3.0)	2.7 (2.4–2.9)
11:15 a.m.	8.4 (2.1–14.7)^#,^*^,^**	3.9 (2.4–5.4) ^#,^*^,^**	66.5 (62.5–70.1)*	69.5 (65.9–73.1)	2.7 (2.4–3.0)	2.6 (2.3–2.8)

Values represent mean values and 95% confidence intervals

^#^*p* < 0.05 as compared to morning urine

*p < 0.05 as compared to 7:15 a.m.

**p < 0.05 as compared to 9:15 a.m.

#### aMT6s/crea

The average aMT6s/crea baseline values in both light groups were comparable, with 20.8 (CI 12.2–29.4) ng/mg in the OL group and 14.0 (CI 8.6–20.5) ng/mg in the BL group (*p* = .542); aMT6s/crea changed significantly under both light setups (OL and BL *p* < .001). Pairwise comparisons revealed that changes in both light setups occurred from morning urine to 9:15 a.m. as well as to 11:15 a.m. (*p* < .001) and from 9:15 a.m. to 11:15 a.m. (*p* < .001). In the BL setup, aMT6s values additionally decreased from 7:15 a.m. to 9:15 a.m. (*p* = .015). We did not detect any time-dependent effect of the two light intensities (*p*_1_ = .717, *p*_2_ = .918, *p*_3_ = .654, *p*_4_ = .477, *p*_5_ = .172, *p*_6_ = .608; with *p*_1_: morning urine to 7:15 a.m.; *p*_2_: morning urine to 9:15 a.m.; *p*_3_: morning urine to 11:15 a.m.; *p*_4_: 7:15 a.m. to 9:15 a.m.; *p*_5_: 7:15 a.m. to 11:15 a.m.; *p*_6_: 9:15 a.m. to 11:15 a.m.).

Changes in aMT6s/crea levels from pre- to post-light exposure in subjects with evening chronotype were greater (35.76 ± 24.01) compared to subjects with a moderate chronotype (6.96 ± 14.60; *p* = .002). No differences were found for morning versus evening type subjects (*p* = .072) or morning versus moderate chronotype subjects (*p* = .268).

#### Trp

**Fig. 1:**
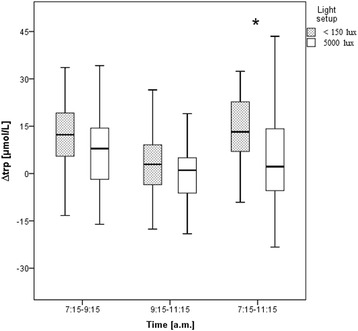
Differences in tryptophan serum levels (*∆*) over time for the two light setups (office light and bright light). *p < 0.05 versus the other light setup

Mean baseline trp levels (at 7:15 a.m.) were 82 μmol/L (CI 77.2–86.9) for OL and 75.6 μmol/L (CI 70.3–81.0) for BL. The trp levels changed over time in the OL setup only (*p* < .001). Changes occurred from 7:15 a.m. to 9:15 a.m. and from 7:15 a.m. to 11:15 a.m. (*p* < .001 at both times). A significant light effect was only found for the period from 7:15 a.m. to 11:15 a.m. (*p* = .045; Fig. 1).

#### Kyn

Mean baseline trp levels (at 7:15 a.m.) of 3.2 μmol/L (CI 2.8–3.7) for OL and 2.8 μmol/L (CI 2.5–3.0) for BL were measured. The kyn levels remained unchanged in both light setups (BL, *p* = .288; OL, *p* = .070). No differences in the scores between different time periods were detected between the two light setups.

### Association between performance and biochemical measures

Correlation analyses revealed that changes in kyn values were significantly associated with changes in TRT (*p* = .032; *ρ* = 0.280), RTCR (*p* = .010; *ρ* = 0.334), CR (*p* = .049; *ρ* = 0.257), and trp values (*p* < .001; *ρ* = 0.645).

## Discussion

Early morning light exposure has been shown to improve a variety of physical and cognitive performance indices after sleep. We evaluated the impact of a single early morning BL exposure on performance and biochemical parameters. In addition to previously published data [20], the novel approach of this study was the quantification of a possible association between the impact of BL on the courses of trp and trp degradation products, melatonin and kyn, and motor performance parameters.

An exclusive effect of the BL intervention was found for trp data only, showing a lower decrease in trp values over time in the BL setup. With respect to the measured sensorimotor functions, participants reacted identically in both light setups, with better balance and choice reaction performance after both light exposures. Some of the changes in performance parameters were found to be associated with changes in biochemical parameters.

### Performance data

According to the suggestion that most prominent [39] and immediate effects [40] of BL exposure on cognitive performance should be expected during the day when provided before midday, an early morning light regimen was chosen in the present design. However, the single early morning light intervention was not effective in increasing visuomotor performance. The present results are also in contrast to those from a study conducted during the Antarctic winter, where BL was effective in improving alertness and cognitive performance [1]. The discrepancies in results are most probably a consequence of differences in study design, the variation in natural light exposure related to the two study locations (Antarctica and Central Europe), and the different cognitive performance tests (Digital Symbol Substitution Test and Single Letter Cancellation Test were used in the study by Corbett et al. [1]). Also, in contrast to our study, 1-hour morning bright light application (1000 lux) resulted in shorter reaction times on the psychomotor vigilance task compared to an illuminance of 200 lux [6]. Moreover, compared to 200 lx, after exposure to 1000 lux, application participants felt less sleepy and more energetic. The authors concluded that morning bright light can improve feelings of alertness and vitality, objective performance, and physiological arousals. In a similar setting, the same research group demonstrated significant improvements under 1700 lux vs. 165 lux (each 90 min exposure time) in difficult but not easy task performances (Backwards Digital-Span-Task, BDST) and no differences in the Psychomotor Vigilance Test (PV) [10].

An increase in reaction latency was posited to be responsible for the elimination of labyrinth performance after BL exposure in a mouse study [41], which could explain the lack of effect of BL on motor performance seen in our study. The decrease in performance was associated with higher levels of corticosterone in blood serum and a higher level of thigmotaxis (behavioral anxiety measure), which were also highly negatively correlated with learning and memory performance. Our group recently reported similar behaviour patterns in humans, who showed impaired sustained attention after short morning BL exposure [20].

Most recent investigations of BL and physical performance focused on endurance and strength-specific performance, showing an enhancing effect of BL [25, 26]. Early investigations reported that, in contrast to 50 lux light exposure, the application of 5000 lux is sufficient to increase handgrip strength, associated with lower rectal temperatures [42]. Suppression of melatonin and associated thermoregulatory changes through BL exposure demonstrates the potential of a “pre-cooling” effect prior to long-lasting endurance exercise [23, 26, 42]. A concomitant increase in individual strain is thought to be an important determining factor in physical performance enhancement as a result of BL exposure [25]. In contrast, in a randomized controlled trial, BL exposure 17 h after the individual midpoint of sleep reduced melatonin levels but failed to improve reaction time or handgrip strength in athletes [22]. In the same setting, evening light exposure enabled top athletes to better maintain performance across a 12-min cycling time trial [43]. Nevertheless, our results indicate a limited effect of BL on sensorimotor skills. In addition to the variations in the circadian phase shift that result from light exposures at different times of the day, the type of physical performance must be taken into consideration; strength and endurance performances are primarily dependent on muscular and cardiovascular parameters, whereas the nervous system predominantly determines visuomotor and sensorimotor performance.

O’Brien and O’Connor [44] found that heart rate, power capacity, and perceived exertion, as well as the oxygen consumption, alertness, and mood of competitive cyclists during an incremental exercise test, were identical in three different light intensities. Performance output measures for supra-maximum intensities were also unaltered in long-distance runners [45], even though higher light intensity reduced glucose and adrenaline levels. This evidence, as well as the present results, leads to the presumption that, with respect to light environment, the human organism reacts in a similar way when it comes to coordinative, cognitive, and endurance performance.

The suggestion that the chronotype of an individual must also be considered when the effect of BL on physical performance is evaluated [25] could only partially be confirmed with the present data (i.e., changes in aMT6s/crea levels from pre- to post-light exposure). In contrast to those previous reports, the chronotype differences in the present study were between the moderate and evening chronotype. The low number of participants and the different study designs (2 h light exposure in the evening/night) may account for the lack of light effect on the remaining parameters. Since the performance tests and the urine collections were done at fixed time points in our study, influences of the individual circadian phases on the absolute values of aMT6 have to be considered. For example, for a volunteer with an evening-chronotype, a wake-up time of 6 a.m. is very early and may have negatively influenced the performance tests.

The BL exposure time might be also a factor contributing to BL-induced changes in performance parameters.

### Biochemical data and their association with performance

The hypothesis of augmented suppression of aMT6s after BL exposure, along with enhanced visuomotor reaction times, cannot be confirmed with the present data. The time course of aMT6s/crea and the missing differences of aMT6s/crea between BL and OL were similar in the same setup by measuring serum melatonin concentrations [20]. Contrary to this, Chellappa et al. [18] found strong correlations between faster reaction times and salivary melatonin concentrations. It was also recently shown [46] that a regimen comprising a trp-enriched diet in combination with BL exposure during the day promoted melatonin secretion at night.

Even if the aMT6s courses did not show any associations with performance parameters in the present study, correlations between kyn and visuomotor performance parameters were found. These findings indicate that the kyn pathway of trp degradation could influence certain aspects of visuomotor performance, emphasizing the potential and importance of their implementation in future research on BL and performance. Trp is to a minor degree metabolized to serotonin and subsequently converted to melatonin, while trp is degraded within the so-called kyn pathway to kyn to a major degree [40]. Up to now, only a few studies on the possible effects of plasma trp on cognitive functions and exercise performance in healthy subjects have been published. Most studies focused on trp depletion or supplementation either in healthy volunteers or in patients with psychiatric diseases. In healthy individuals, acute trp depletion reduced the perception of facial emotions during OL exposure, which was not detected during BL application [29]. A trp-enriched diet on the antecedent evening was successful in increasing morning alertness and attention in subjects with mild sleep complaints [47]. In the setting of acute and chronic diseases, however, dietary intake of trp may improve a variety of neuropsychological functions [48, 49]. Two hours of BL exposure resulted in a small increase in subjective wellbeing (affective state) in healthy subjects who were being subjected to relatively unpleasant conditions; OL was associated with a corresponding reduction in these parameters. Under both conditions, serum trp was reduced, indicating no light-specific effect on trp [30].

Simple and effective strategies for enhancing cognitive and physical performance are desirable in the working world, as well as in competitive sports. BL has previously been shown to have the potential to meet these demands. In the present study, we were not able to prove BL’s performance-enhancing properties. Nevertheless, the evaluation of trp degradation pathways revealed some interesting information regarding the effect of a single, short BL exposure on biochemical data; the fact that trp degradation was affected by the BL regimen emphasizes that the concept of including trp and its metabolites in BL research is a desirable and appropriate approach.

### Limitations

One limitation of our study was that situations outside the protocol were not controlled for light. Low intensity light is physiologically important [50, 51], and the possibility cannot be excluded that BL application immediate after waking would have resulted in more pronounced differences between BL and OL. Exposure time to BL might also be a factor contributing to BL-induced changes in performance parameters. A dose-response relationship between the duration of BL light exposure and cycling performance was recently shown [52]. Thus, we cannot exclude the possibility that a longer exposure duration than the 30 min of BL applied in our study would have resulted in more significant differences between the BL and OL experiments.

The analysis of urinary sulfatoxymelatonin (aMT6s/crea) has some limitations since the absolute values are dependent on the time length of urine pooling, and the statistical comparison between time courses of aMT6/crea with serum kynurenine and tryptophan was not possible. However, the aMT6s/crea changes in OL and BL were similar to those found in serum melatonin in previously published data from the overall research project [20].

Elimination of a first-order effect was attempted by performing a test phase before each performance trial. Nevertheless, the observed results suggest the possibility that a light-dependent effect was potentially confounded by a training effect, substantiated by increased performance after both light exposures, which was independent of light intensity.

## Conclusion

Our results demonstrate that application of bright light in the morning hours has a limited effect on visuo- and sensorimotor performance. Tryptophan degradation pathways in the morning show diverse courses after office light and bright light exposure. This suggests that tryptophan can potentially be altered by bright light exposure.

## Abbreviations

ANOVA: Analysis of variance
BL: Bright light
CI: Confidence interval
CR: Correct reaction
FR: False reaction
HPLC: High-pressure liquid chromatography
KYN: Kynurenine
LED: Light-emitting diode
MEQ: Morningness-Eveningness Questionnaire
OL: Office light
PSQI: Pittsburgh Sleep Quality Index
RTCR: Reaction time of correct reaction
RTFR: Reaction time of false reaction
SD: Standard deviation
TRP: Tryptophan
TRT: Total reaction time
